# Successful Salvage Treatment With Fostamatinib for an Adolescent Patient With Refractory Chronic Immune Thrombocytopenia

**DOI:** 10.7759/cureus.91569

**Published:** 2025-09-03

**Authors:** Masaki Nishizawa, Kenichi Sakamoto, Shoji Saito, Yozo Nakazawa

**Affiliations:** 1 Pediatrics, Shinshu University School of Medicine, Matsumoto, JPN

**Keywords:** adolescent medicine, chronic immune thrombocytopenia, fostamatinib, growth plate dysplasia, immune thrombocytopenia (itp)

## Abstract

Fostamatinib (FOS) is a novel drug for chronic immune thrombocytopenia (cITP) that inhibits spleen tyrosine kinase (Syk) signaling. FOS is approved for the treatment of cITP in adults but has not been approved for pediatric and adolescent patients with cITP. We present the first case of refractory cITP in an adolescent patient who was successfully treated with the addition of FOS to prednisolone and thrombopoietin receptor agonists. Although bone dysplasia requires careful monitoring, FOS is effective in the treatment of adolescent cITP refractory to conventional therapies and should be administered on a risk and benefit.

## Introduction

Fostamatinib (FOS) is a novel drug for chronic immune thrombocytopenia (cITP) that inhibits spleen tyrosine kinase (Syk) signaling, resulting in the suppression of phagocytosis and destruction of platelets bound to antiplatelet autoantibodies by macrophages. FOS is approved for adults by the Food and Drug Administration (FDA) and Japan (Ministry of Health, Labour and Welfare (MHLW)) based on the results of a phase III clinical study, and its long-term safety and efficacy have been demonstrated [1-3]. In the large real-world data from Spain, the efficacy and safety profile of 138 adult patients with ITP receiving FOS was reported. The patients enrolled this study were heavily treated ITP before the administration of FOS (the median number of therapies before FOS was four). A total of 109/138 (79.0%) patients obtained a platelet response (platelet count of >30,000/μL), and median time to platelet response was 11 days. The adverse effect due to FOS were mainly mild (diarrhea 28/139, and hypertension 21/139) and only one patient developed venous thromboembolism and acute myocardial infarction [4]. In Japan, FOS is approved for the treatment of cITP in adults (>18 years old) but has not been approved for pediatric and adolescent patients with cITP[1] . This is because preclinical studies in juvenile rats or rabbits treated with a higher dose of FOS than the approved dose showed an increased risk of bone dysplasia. We present the first case of refractory cITP in an adolescent patient who was successfully treated with the addition of FOS to prednisolone (PSL) and thrombopoietin receptor agonists (TPO-RAs). The addition of FOS reduced PSL and TPO-RA doses and decreased bleeding events.

## Case presentation

**Figure 1:**
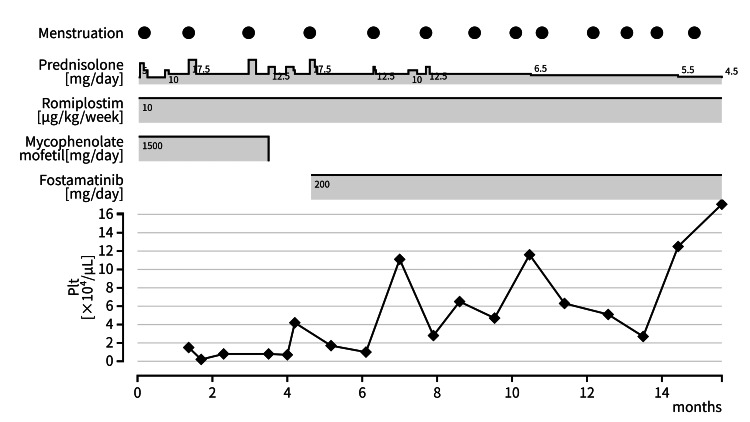
The patient’s clinical course before and after fostamatinib administration The black circles indicate menstruation, and the numbers in the figure represent the dosage of each of the drugs. The solid line represents platelet counts.

A 12-year-old girl had been diagnosed with ITP when she was nine years old. She presented with subcutaneous bleeding and epistaxis, and her blood tests revealed severe thrombocytopenia (11,000/μL) without any abnormality of white blood cells, anemia, or coagulation abnormalities. No abnormal cells were seen and the number of megakaryocytes in her bone marrow was found to be increased. There were no findings suggestive of collagen disease, with negative results for various autoantibodies. These results are consistent with the diagnosis of ITP. She received intravenous immunoglobulin and PSL, which temporarily increased her platelet count; however, reduction in PSL dosage led to a decrease in platelet count (<20,000/μL) and the recurrence of bleeding symptoms. The PSL dosage could not be tapered even at six months since the ITP diagnosis, and TPO-RA (eltrombopag) was added for cITP. However, the therapeutic effect of eltrombopag was insufficient, so we switched from eltrombopag to romiplostim. Furthermore, rituximab and mycophenolate mofetil were subsequently added, which all failed to induce a sufficient increase in platelet count of >20,000/μL with the maximum dosage of TPO-RA (romiplostim; 10 μg/kg/week) and PSL (4 mg/day (≒0.1 mg/kg)). Since a reduced dose of PSL would reduce platelets to less than 10,000/μL and difficult to access medical centers, we continued to administer low doses of PSL. Owing to this, she experienced worsening bleeding symptoms and anemia (hemoglobin level 7.7 g/dL) due to menstruation for approximately three years from onset. At every appearance of bleeding symptoms, including menstruation, we increased the PSL dosage to 1-2 mg/kg/day for several days. At the 12 years of age, three years after the initial onset, she began showing signs of growth impairment (-1SD) due to long-term PSL therapy, as well as decreased quality of life (QOL) requiring exercise restrictions to avoid bleeding events. To avoid adverse events associated with cITP treatment and to increase QOL, we decided to initiate FOS at 200 mg/day after obtaining approval from the institutional ethics committee (approval number B0961). We decided not to perform a splenectomy because we were concerned about the risk of infection and the need for long-term antibiotic prophylaxis. After more than six months from the first menstrual period, we carefully explained the risk of growth plate dysplasia, which was an emerging adverse effect of FOS and obtained informed consent for FOS administration from the patient and her guardian. The key laboratory findings at the start of FOS are shown in Table 1. After initiating FOS, her platelet counts gradually increased, and the frequency of bleeding symptoms and menstrual blood loss decreased. At the last follow-up (nine months after FOS initiation), her platelet count had been maintained above 40,000/μL. We were able to taper the daily PSL dose, and increasing the PSL dosage during menstruation was no longer required. No adverse events, including hypertension, liver function abnormalities, diarrhea, nausea, and growth plate dysplasia associated with FOS, were observed at the last follow-up. We have described the patient’s clinical course before and after FOS administration (Figure 1). The black circles indicate menstruation, and the numbers in the figure represent the dosage of each of the drugs. The solid line represents platelet counts).

**Table 1:** Key laboratory findings at the start of fostamatinib

Laboratory findings	Results	Reference range
White blood cells (/μL)	9,840	3,800-10,100
Hemoglobin (g/dL)	12.3	11.9-14.9
Platelet count (/μL)	1000.0	180,000-440,000
Aspartate aminotransferase (U/L)	11	13-33
Alanine aminotransferase (U/L)	11	7-23
Total bilirubin (mg/dL)	0.46	0.4-1.5
Alkaline phosphatase (U/L)	107	105-483
Lactate Dehydrogenase (U/L)	220	145-270
Creatinine (mg/dL)	0.52	0.4-0.66
Blood urea nitrogen (mg/dL)	10.3	6.8-19.2
Anti–nuclear antibody	Negative		
Anti–dsDNA antibody	Negative	

## Discussion

In this adolescent case, despite resistance to multiple lines of therapy, rapid platelet count improvement was achieved with FOS. Recent progress in understanding the pathogenesis of ITP and treatment strategies has reduced the number of bleeding events and improved quality of life (QOL) in patients with cITP. However, all novel drugs, such as Syk inhibitors, neonatal Fc receptor inhibitors, and Bruton’s tyrosine kinase inhibitors, have been approved only for adult patients with cITP and not for pediatric and adolescent patients [5]. In Japan, the approved second-line treatments for pediatric patients with cITP are TPO-RAs and rituximab, highlighting the unmet need for alternative treatments due to resistance to these treatments.

The management of cITP in children and adolescents remains challenging, particularly in those who fail to respond to corticosteroids, TPO-RAs, rituximab and immunosuppressive agents in Japan. A splenectomy is considered for refractory cases, but concerns about the risk of long-term infection limit its use in children and adolescents. Therefore, the novel targeted small molecule inhibitors such as FOS may provide potentially useful alternative strategies.

While FOS is one option of salvage treatment for cITP, clinical trials for children and adolescent have not been conducted, and preclinical safety data reported adverse effect on actively growing bones, including abnormal cartilage formation in the femoral head and growth plate dysplasia in the femur and femoro-tibial joints in juvenile rat or rabbit treated with 30 mg/kg and 60 mg/kg of FOS, which were estimated as 7.5−15 times higher than the approved dose [6]. Although the safety profile of the FOS for adolescents at the approved doses administered in clinical practice has not been studied, it is important that no adverse skeletal effects, such as abnormal cartilage formation in the femoral head or growth plate dysplasia in the femur and femoro-tibial joints, occurred in our cases over a 9-month period.

The safety profile of the FOS for children and adolescent at the doses administered in clinical practice has not been studied. Although bone dysplasia requires careful monitoring, FOS is effective in the treatment of adolescent cITP refractory to conventional therapies and should be administered on a risk and benefit. Prospective clinical studies for adolescent cITP are urgently needed to determine the efficacy and optimal dosing of the new agent including FOS for cITP, as well as to establish safety monitoring protocols.

## Conclusions

This case report describes a 12-year-old with cITP refractory to multiple therapies, who showed sustained platelet improvement and reduced bleeding after FOS addition, without adverse events over nine months. The treatment using FOS for cITP is off-label and should be started after obtaining institutional ethics committee approval and written informed consent from both the patient and their guardian. Although not approved for pediatric use and requiring caution for growth-related risks, FOS may be a promising option for treatment-resistant cITP in adolescents.
